# Development of an ultrasound-assisted pre-treatment strategy for the extraction of d-Limonene toward the production of bioethanol from citrus peel waste (CPW)

**DOI:** 10.1007/s00449-023-02924-y

**Published:** 2023-09-23

**Authors:** George Mbella Teke, Liza De Vos, Isle Smith, Tamryn Kleyn, Zwonaka Mapholi

**Affiliations:** Department of Chemical Engineering, Stellenbosch University, Private Bag X1, Matieland, Stellenbosch, 7602 South Africa

**Keywords:** Ultrasound-assisted extraction, Pretreatment, Citrus peel waste, d-Limonene optimization, Bioethanol production

## Abstract

Citrus is one of the world’s most abundant fruits containing vitamins, pigments, and fragrances, making it vital for several industries. However, these fruits contain about 45–50% residues (peels), which often end up as waste and can be harmful to the environment if not properly treated. Bioethanol production from citrus peel waste offers a potential solution to this problem. Hence, this study explores the potential of using ultrasound-assisted pre-treatment method as a novel strategy to extract d-Limonene (essential oil in the residue), and further demonstrates bioethanol production. This was done by investigating ultrasonication’s optimal effect on pre-treatment of the citrus residue, followed by bioethanol production. The results show that, optimum values for d-Limonene extraction were obtained at a temperature of 14.6 °C and an ultrasound intensity of 25.81 W/cm^2^ with a validation yield of 134 ± 4.24 mg/100 g dry CPW. With optimal ultrasonic parameters, the study went further to demonstrate the effect of the essential oil on bioethanol production which is hindered by the oils present. Key findings show better bioethanol yield once the essential oil was extracted (treated) from the citrus waste as opposed to it not extracted (untreated), with a 66 and a 29% increase when comparing simultaneous saccharification and fermentation (SSF) and sequential hydrolysis and fermentation (SHF) respectively. Based on this result, ultrasound-assisted extraction as a pretreatment method was found suitable for bioethanol production from citrus residue and could be utilized as a biorefinery pre-treatment approach to scale bioethanol production.

## Introduction 

Citrus is one of the world’s most abundant crops, with an estimated annual production of approximately 102 million metric tons in 2007 [1, 2]. The citrus fruit consists of 40 species resulting from several genetic modifications [2] and is characterized by small trees and large shrubs [3]. These characteristics result from their cultivation in Mediterranean countries (in Europe), North Africa, America, Australia, South Africa, and in the tropical and subtropical areas (South East Asia) [4].

Citrus fruit contains a high concentration of vitamin C, making it a vital product in the processing industry as it is widely used in the production of nutrient-dense drinks and beverages. However, 45–50% of its weight is pure juice, with the remainder being considered residues: the peel (flavedo–27%), the pulp (albedo and endocarp–26%), seeds (2%), and others below quality requirements increase this percentage, of about 120 million tons annually [4, 5]. A significant amount of this citrus fruit residue is dumped on nearby landfills or rivers or burned, resulting in environmental pollution and reduced dissolved oxygen in contaminated water [6]. In addition, this method of waste management also causes significant degradation of soil qualities in the surrounding area [4, 5, 7]. Consequently, alternative measures to circumvent these issues while increasing profits for citrus fruit industries would be valorizing the citrus feedstock into producing value-added products (e.g., biofuels, methane, and hesperidin).

In the focus of biofuel production, citrus waste falls into the second-generation feedstock category, which is technically suitable for bioethanol production but not well established industrially for huge production volumes even when they are not food-competitive [8]. Hence, citrus waste is a potentially promising feedstock to increase bioethanol production. However, this has been hampered by the presence of the essential oils found in the waste. During fermentation of citrus waste, the oils accumulate in the membrane of the micro-organism, penetrate through the cell wall, diffuse across the cytoplasmic membrane, and ultimately permeabilize them. The fluidity of the membrane of the microorganism is altered by permeabilization which disrupts the cell structure, leading to the release of the contents of the cell. The cytotoxic effects of citrus waste’s essential oils on microbes prevent fermentation because of the apoptosis and necrosis that results [9].

To overcome this challenge, pre-treatment methods can be employed to reduce the essential oils from the citrus waste before they become soluble with the aqueous fermentation media. Most pre-treatment methods are either thermo-physical or thermochemical processes, making them either very energy-intensive or leading to a reduction of sugars or causing a modification of volatile molecules [10]. Traditionally, these pre-treatment methods (Soxhlet extraction, hydro-distillation, and cold pressing) are good, but their drawbacks (low yield, slow process, loss of polar components, and long extraction times) necessitate novel extraction techniques.

Over the years, novel extraction methods have been developed to solve low yield issues and short extraction times. These methods could either be done in *in situ* or *ex situ* during fermentation. For *in situ* extraction using supercritical fluid, adequate understanding of fluid mass transfer properties is needed, and difficulty in controlling operating parameters is also essential, limiting its use in specific fields [11, 12]. However, novel bioreactor configurations exist, which could help solve such problems from a broad perspective, although mass transfer kinetics is required for their utilization [13–18].

For *ex situ* extraction processes, such as microwave-assisted extraction and ultrasound-assisted extraction, the former is good due to short production time, low energy, and solvent consumption but not efficient when the solvent or target compounds are non-polar or volatile [12, 19, 20], while the latter is more efficient as it does not rely on high temperatures, meaning that the integrity and the concentration of both sugars and essential oils are preserved. This method is also more environmentally friendly since it does not require harsh solvents to extract the essential oils. These traits result from the ultrasound’s mechanical effects that disrupt the cell walls, facilitate fluid mass transfer, and improve the solvent’s penetration into the cells [21].

From the above understanding and to the best of the authors' knowledge, the presence of essential oil in citrus peel waste hinders bioethanol production during fermentation. However, should yield increment be necessary toward implementing a biorefinery valorizing technology of citrus peel waste, pre-treatment should be necessary. Hence, this study aims at optimizing ultrasonication as a potential pretreatment strategy that could be implemented to curb the presence of essential oils in the citrus peel waste while demonstrating bioethanol production. To implement this, an ultrasound-assisted extraction process as a pre-treatment methodology for optimum d-Limonene extraction from citrus peel waste was optimized, and the optimum parameters used to demonstrate the production bioethanol production using *Saccharomyces cerevisiae (S. cerevisiae)*.

## Materials and methods 

### Chemicals and equipment

Peptone, D-( +)-glucose, yeast extract, YPD media, 2-pentanol, citric acid monohydrate, tri-sodium citrate di-hydrate, 99% hexane, and 98% d-Limonene were obtained from Sigma-Aldrich, South Africa. The enzyme cocktail, Viscozyme^®^ was obtained from Novozyme. The yeast (*S. cerevisiae*) was obtained from Anchor Yeast, South Africa.

The equipment used for ultrasound generation is a 24 kHz UP200St ultrasound system. It has a maximum power output of 200W with a maximum amplitude of 190 µm. This is produced by a horn tip transducer of surface area equivalent to 0.38 cm^2^ and a 12 ml flow cell, manufactured by Hielscher, Germany. The bioreactor system used is a product of Glaschem, South Africa. It consisted of a control system, a 1L glass reactor equipped with temperature and pH probes, a stirrer, and a heating jacket connected to a temperature-controlled water bath. Analytical equipment used was the microplate reader ELx800 (Bio Tek Instruments) and UV–Vis spectrophotometer in the Department of Chemical Engineering, Stellenbosch University. Other pieces of equipment used were a heating block, a hot plate, and a halogen lamp-type moisture analyzer manufactured by KERN DERBS.

### Citrus peel waste preparation

Citrus peel waste (CPW) from clementine citrus (*Citrus clementina*), was obtained from a local citrus farm (Babylonstoren, South Africa). After collection, the material was coarsely blended (with Nutribullet Blender) to particle sizes between 2 and 5 mm in size, mixed, and stored at −20 °C prior to use. Before extraction, the required volume was subsequently thawed and further blended into fine pulp with the help of a NutriBullet blender. The samples were then moisture-analyzed (measurements were carried out in triplicates) at 180 °C using a halogen lamp moisture analyzer. Due to the consideration of pumping through the ultrasound system, the solid content was corrected to 4% (w/v) through dilution with deionized water.

### d-Limonene extraction procedure 

**Fig. 1:**
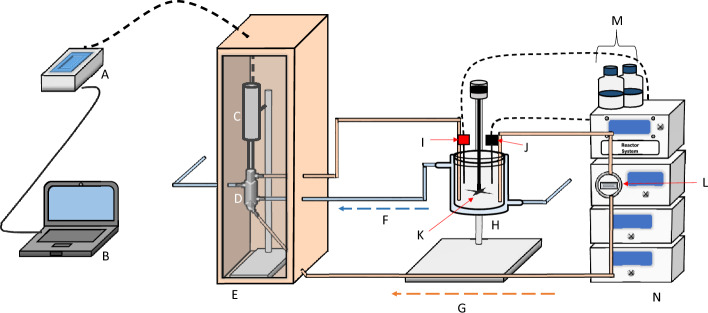
Schematic representation of the ultrasound-assisted extraction set-up (batch recirculation mode). **A** ultrasound generator and control, **B** Laptop, **C** transducer connected to a probe, **D** Jacket ultrasound flow cell, **E** soundproof box, **F** cooling/heating water recirculated from temperature control water bath, **G** material recirculation pipe, **H** jacketed bioreactor, **I**- thermocouple, **J** pH probe, **K** the stirrer, **L** peristaltic pump, **M** acid and base buffer solutions, **N** reactor control system

Extraction of d-Limonene was done in a batch bioreactor that was connected to an ultrasound flow-through system, as depicted in Fig. 1. To carry out the extraction, the reactor was first loaded with 500 g of CPW material at 4% (w/v) solids loading. Thereafter, the material’s temperature was adjusted to the required optimization level using a water bath connected to the reactor jacket and then circulated between the bioreactor and the ultrasound flow cell using the peristaltic pump at 105 ml/min (see Fig. 1 below). This temperature was varied between 14.6 and 85.4 °C and maintained with a re-circulating water bath system. Similar to the temperature, the ultrasound intensity was varied between 1.62 and 49.92 W/cm^2^. The pH was observed to fluctuate between 3 and 4 throughout the experiments but was not controlled. Each experiment was carried out over 60 min with samples taken every 10 min intervals (including 0 min) for GC–FID analysis.

### CPW Fermentation procedure 

Two different fermentation procedures were employed, sequential hydrolysis and fermentation (SHF) and simultaneous saccharification and fermentation (SSF). The enzyme used for hydrolysis, Viscozyme^®^, is an enzyme complex with activities toward the degradation of pectin, hemicellulose, and cellulose with a declared activity of 100 FBG/g, dosed at 1% (mg enzyme/g CPW). Specifically, for pectin degradation, the enzyme complex contains pectin lyase, polygalacturonase, and pectin methylesterases. Also, it contains arabanases, xylanases, and mananases (responsible for hemicellulose degradation), and, β-glucanase and rhamnogalacturonase (responsible for cellulose degradation). The hydrolysis aspect of the SHF procedure was performed in a 250 ml (loaded with 180 g CPW slurry of 5 wt% solids) baffled flasks for 6 h at 50 °C in a shaking incubator at 150 rpm with a controlled pH of 5 (as seen from other studies [22]) using a citric acid buffer. Then, hydrolysates were filtered and pasteurized in a controlled water bath at 70 °C for an hour (then plated on agar to confirm media sterile hold) in preparation for fermentation. After hydrolysis, fermentation was performed with *S. cerevisiae* at 30 °C for 48 h in a shaking incubator at 150 rpm, and samples were taken for HPLC analysis.

Unlike the SHF, 180 g of CPW slurry (consisting of 5wt%) was pasteurized in a control water bath at 70 °C for 1 h, then, simultaneous saccharification and fermentation were performed using *S. cerevisiae* in a 250 ml baffled flask for 48 h, at 37 °C, and pH of 5 and a shaking incubator set at 150 rpm with the recipe from Mark et al., 2007 [23]. These experiments were all carried out in triplicate runs with sample quantification by HPLC analysis.

The yeast (*S. cerevisiae*) used for fermentation was obtained from Anchor Yeast, Cape Town, and kept at 4 °C until use. Before pre-culturing, the cells were prepared in a modified YPD (yeast extract–peptone–dextrose) media (consisting of yeast extract (10 g/L), peptone (20 g/L), and dextrose (20 g/L)) from Choi et al., 2013 [24], then sterilized using an autoclave at 121 °C for 15 min.

### Experimental design

#### Pre-treatment (extraction of d-Limonene)

The extraction of d-Limonene was optimized using two factors and five-level central composite design (CCD). The two independent factors were temperature (X_1_, °C) and ultrasound intensity (X_2_, W/cm^2^). The ranges of the independent variables are based on typical literature values, with consideration of the physical limits of the ultrasound equipment as well as the plausible scope of operation for pilot and industry-scale ultrasound extractions. The response variable is the yield of d-Limonene (mg d-Limonene /100 g of CPW). The full experimental design matrix is illustrated in Table 1.

**Table 1:** Complete central composite design matrix for two factors at five-level settings used for the optimization of the ultrasound pre-treatment of CPW to recover essential oils, d-Limonene

Factors	Levels				
Coded levels	−α	−1	0	1	+ α
Parameters Temperature (X_1_,°C)	Actual values				
	14.6	25	50	75	85.4
Ultrasound intensity (X_2_, W/cm^2^)	1.62	8.69	25.77	42.93	49.92

The optimal conditions for ultrasound operation for the removal of d-Limonene were determined through desirability and response surface methodology (RSM). The response surfaces regression was carried out by fitting experimental data on a second-order polynomial, given by Eq. (1) below

1 $${Y}_{o}={\beta }_{o}+\sum_{i=1}^{4}{\beta }_{i}{X}_{i}+\sum_{i=1}^{4}{\beta }_{ii}{{X}_{i}}^{2}+\sum_{i=1}^{4}\sum_{j=i+1}^{4}{\beta }_{ij}{X}_{i}{X}_{j}$$

where ${Y}_{o}$ represents the response variable. *β*_*o*_*, β*_*i*_*, β*_*ii*_, and *β*_*ij*_ represent the regression coefficients for intercept, linear, quadratic and interaction respectively; *X*_*i*_ and *X*_*j*_ represent two independent variables, where i ≠ j. Model regression, RSM and desirability were all carried out using Statistica (Version 13.3, TIBCO Software, USA).

### Analytical assays

#### GC–FID for d-Limonene quantification 

25 g samples collected at the end of extraction were centrifuged at 2000 rpm for 10 min. The liquid phase was then separated followed by the addition of hexane to extract non-polar d-Limonene from the aqueous phase to the hexane solution. An analytical standard ratio of 1:0.2 of liquid citrus peel waste to hexane was used. The two-phase liquid was shaken vigorously for 30 s and then placed in a vortex shaker for 1 min. The liquid was then centrifuged at 1500 rpm for 5 min, after which the hexane phase was collected. 1.5 ml of hexane phase was mixed with 15 μl of 2-pentanol, 2-pentanol being the internal standard. GC-FID was used for d-Limonene quantification. The yield of d-Limonene was then calculated based on the concentration of d-Limonene in the hexane phase per 100 g of initial dry CPW as:

2 $${\text{Yield~of~d}} - {\text{limonene}}~ = ~\frac{{{\text{mass~of~d}} - {\text{limonene}}~}}{{100~g~{\text{of}}~{\text{dry~CPW}}}}$$

#### HPLC for ethanol quantification 

A high-performance liquid chromatography (HPLC) system equipped with a Shodex 101 reflective index detector was used to measure the concentration (in g/L) of ethanol. The HPLC calibration standard for ethanol was produced, and the analysis was performed on a Biorad HPX-87H column with a 250 × 7.8 mm fitted guard cartridge. The column was operated at 65 ºC, and the mobile phase consisted of 0.005 M sulfuric acid flowing at 0.6 ml per minute.

### Statistical analysis

Randomized CCD experiment data were analyzed using Statistica (version 13.3, TIBCO, USA), by RSM. The experimental design utilized the CCD, where the influence of the process variables on extraction and their possible interactions were assessed using analysis of variance (ANOVA) and *F*-test. Significant differences between means were assessed using one-way ANOVA (Tukey HSD test). Significant differences are defined as *p-value* is less than 0.05 (*p* < 0.05). Results are stated as means ± standard deviations. Triplicate (*n* = 3) means measurements were performed.

## Results and discussion

### Results

#### Optimization of citrus peel waste pre-treatment (recovering d-Limonene) by ultrasound

The pre-treatment experimental results generated by employing two factors (temperature and ultrasound intensity) at five levels of central composite design are summarized in Table 2. ANOVA based on the results of Table 2 was employed to evaluate and fit a second polynomial equation that describes the empirical relationship between the yield of d-Limonene and the independent variables. The results of the ANOVA are presented in Table 3 below. From the ANOVA results, the second-order polynomial equation is presented by Eq. (3) below:

**Table 2:** Central composite design matrix and the response values for the actual and the predicted yield for d-Limonene at the endpoint of extraction (60 min)

Exp	Temperature (°C)	Ultrasound Intensity (W/cm^2^)	d-Limonene yield (mg/100 g dry CPW)	Predicted d-Limonene yield (mg/100 g dry CPW)
1	25	8.69	132.93	102.69
2	25	42.85	105.47	77.94
3	75	8.69	72.57	47.13
4	75	42.85	16.81	22.40
5	14.6	25.77	107.46	141.36
6	85.4	25.77	59.84	62.72
7	50	1.62	8.97	40.71
8	50	49.92	1.83	5.75
9 (C)	50	25.77	24.99	23.23
10 (C)	50	25.77	20.94	23.23

**Table 3:** Analysis of variance and regression coefficients for the second-order polynomial model in terms of actual variables for d-Limonene

	d-Limonene yield (60 min)		
Coefficient	Effect estimate	*t*-Ratio	*P*-value
Intercept			
βo	22.87	11.29	0.0562
Linear			
β1	−55.54	−27.43	0.0232*
β2	−24.73	−12.20	0.0520
Quadratic			
β11	78.88	29.46	0.0216*
β22	0.63	0.23	0.8530
Interaction			
β12	−16.84	−5.88	0.1072
Model	df (SS)	*F*-value	*P*-value
Lack of fit	3 (4298.62)	174.71	0.05554
R^2^	0.79		

3 $$Y=22.87-55.54{X}_{1}-24.73{X}_{2}+78.88{X}_{1}^{2}+0.63{X}_{2}^{2}-16.84{X}_{1}{X}_{2}$$

From the results of the ANOVA on the yield of d-Limonene recovered, the model coefficient of determination, R^2^ was found to be 0.79. The high R^2^ (0.79) indicates that the model has adequate precision in describing the relationship between the yield of d-Limonene and the independent variables (temperature and ultrasound intensity). R^2^ of 0.79 further implies that 21% of total variation is not explained by the model. Models with R^2^ > 0.7 are generally acceptable but should be in conjunction with lack of fit, *F*-values, sum of squared estimate of errors (SSE), and residual plots as shown in literature in such scenarios [25–29]. The model *F*-value and the* p*-value were 174.71 and 0.05554 respectively. The large *F*-value and the* p*-value > 0.05 indicate that the model had an insignificant lack of fit, revealing that the model has good adequacy.

#### Impact of pretreatment parameters on the yield of d-Limonene

**Fig. 2:**
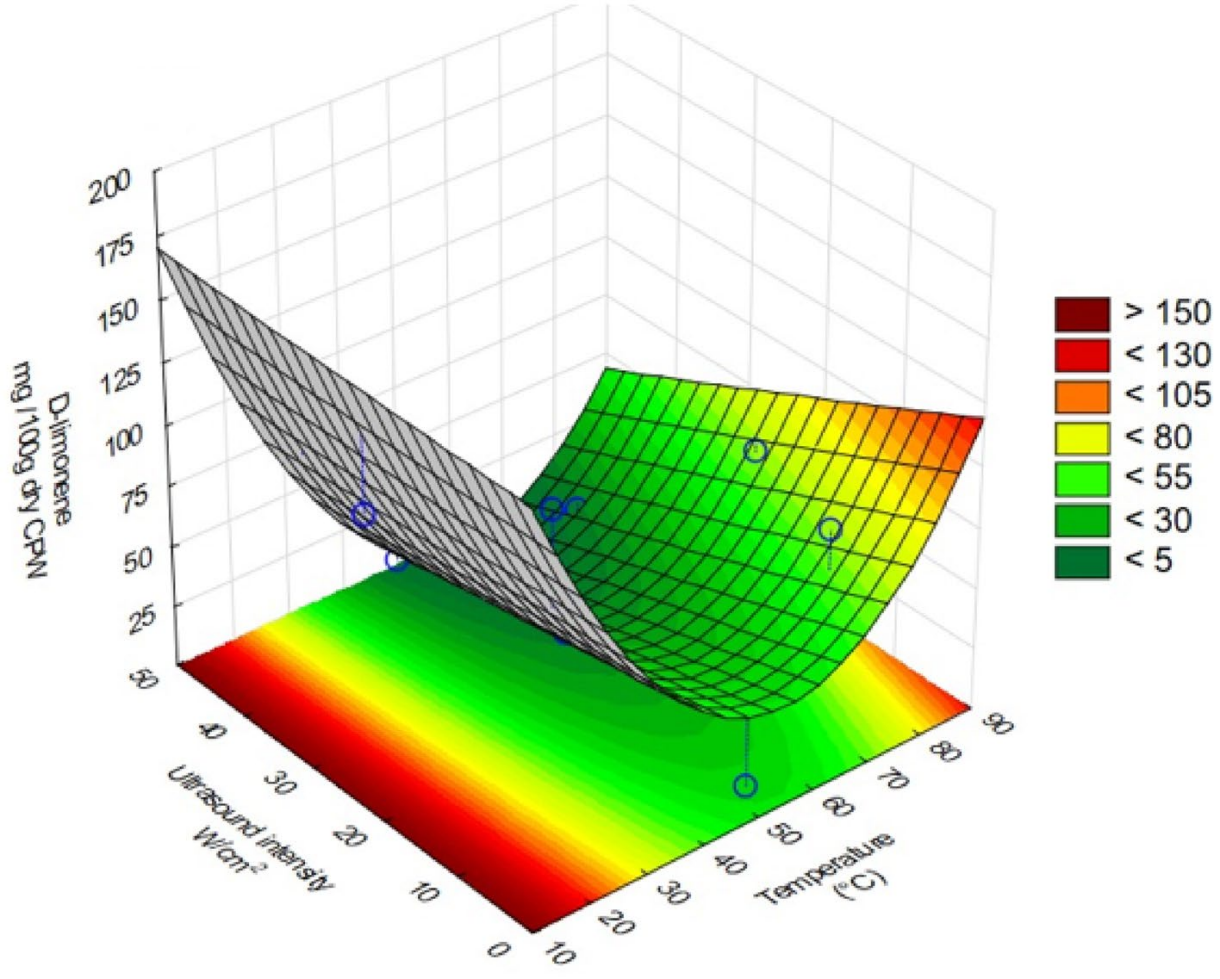
Response surface illustrating the effects of pre-treatment temperature and ultrasound intensity on the yield of d-Limonene recovered from the citrus peel waste

The influence of the pre-treatment parameters on the endpoint yield of d-Limonene was evaluated through ANOVA (Table 3) and was illustrated graphically through the response surface plot (Fig. 2). The results reveal that both linear (X_1_) and quadratic (X_1_^2^) effects of temperature were significant (*p*-values < 0.05). The linear (X_2_) and the quadratic effects (X_2_^2^) of ultrasound as well as the interaction effect between temperature and ultrasound (X_1_X_2_) were found to be insignificant. However, the linear effect of ultrasound (X_1_) and the intercept coefficient (β_0_) are slightly insignificant (*p*-value ~ 0.05). Therefore, it is crucial to consider these factors when reducing the model.

Temperature had the highest impact on the yield, as the magnitude of the effect of both linear (β_1_ = −55.54) and quadratic (β_11_ = 78.88) terms were greater than the magnitudes of ultrasound (β_2_ = −24.73 and β_22_ = 0.63). The negative linear coefficients suggest that the yield of d-Limonene decreases with increasing levels of either temperature or ultrasound intensity. Similarly, positive quadratic coefficients for temperature (β_11_ = 78.88) and ultrasound intensity (β_22_ = 0.63) reveal that the yield decreases with increasing temperature or ultrasound intensity. However, it also reveals that curvature exists within the experimental domain, after which the yields increase with increasing factors levels (trends visible in Fig. 2).

**Fig. 3:**
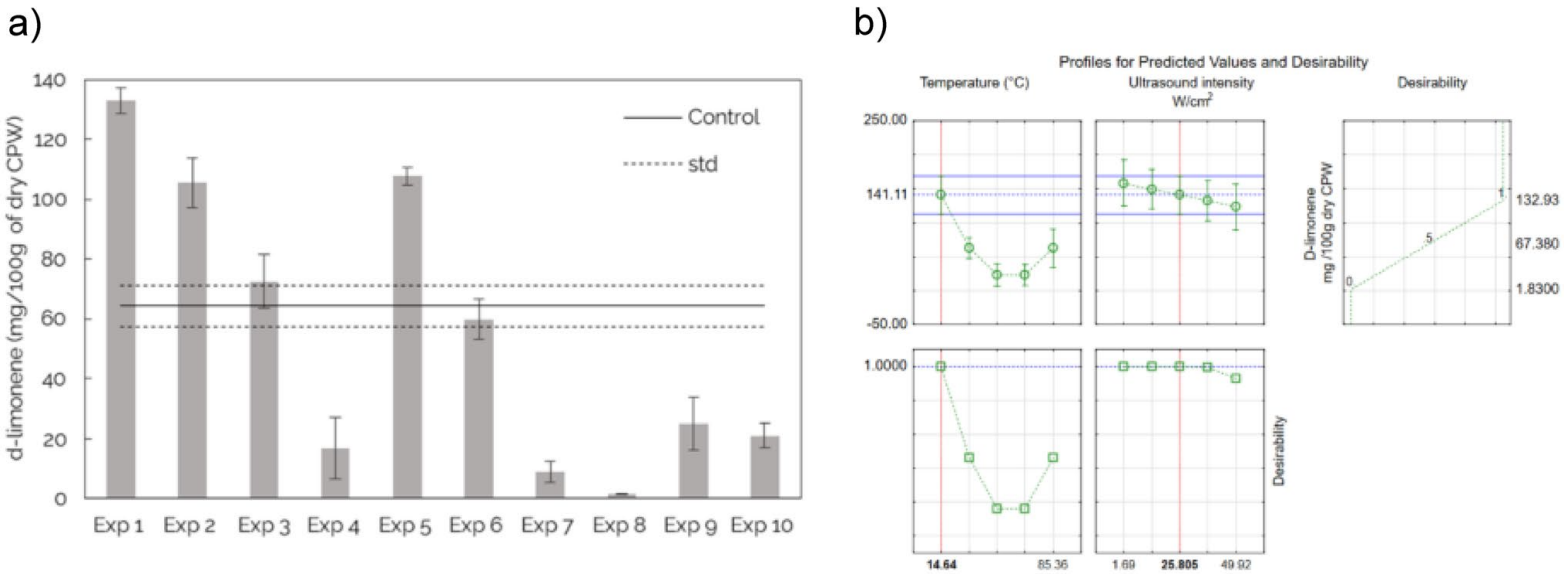
**a** Comparison of the yield of d-Limonene recovered across the experimental runs of the central composite design compared to a control run conducted at 0 W/cm^2^ ultrasound intensity and temperature of 50 °C at 60 min treatment time. **b** Desirability profile for identifying the optimum levels of temperature and ultrasound intensity for removal of d-Limonene from CPW based on the reduced model

Overall, the yield of d-Limonene recovered within the experimental domain ranged from 1.83 mg/100 g of dry CPW (50 °C, 49.92 W/cm^2^) to 132.93 mg/100 g of dry CPW (25 °C, 8.69 W/cm^2^). The influence of temperature has been confirmed by statistical significance through ANOVA, to further investigate these effects, and the influence of temperature at specific ultrasound intensities was investigated. At constant ultrasound intensity of 8.69 W/cm^2^, it was found that the yield of d-Limonene recovered at 25 °C was 132.93 mg/100 g of dry CPW and at 75 °C was 72.57 mg/100 g of dry CPW (shown by experiment 1 and 3 in Table 2 and Fig. 3A. Similarly, at a constant ultrasound intensity of 42.85 W/cm^2^, the yields of 105.47 and 11.43 mg/100 g of dry CPW were recovered at 25 and 75 °C respectively (experiments 2 and 4 in Table 2 and Fig. 3A). At an ultrasound intensity of 25.77 W/cm^2^, the yields of 107.46 and 59.84 mg/100 g of dry CPW were recovered at 14.6 and 85.4 ^o^C respectively (experiments 5 and 6). From these results, it can be observed that regardless of the level of ultrasound intensity, lower temperatures resulted in higher yields as compared to higher temperatures. Therefore, lower temperatures are desirable for ultrasound-assisted extraction of d-Limonene.

The results from ANOVA have demonstrated that the effects of ultrasound intensity are slightly insignificant, however, at constant temperatures, the effects of varying ultrasound intensities could still be investigated. At a constant temperature of 25 °C, the yield of d-Limonene recovered declined from 132.93 to 105.47 mg/100 g of dry CPW when ultrasound intensity was increased from 8.69 to 42.85 W/cm^2^ (experiments 1 and 2). At a constant temperature of 75 °C, the yields were found to be 72.57 and 16.81 mg/100 g of dry CPW at 8.69 to 42.85 W/cm^2^ (experiments 3 and 4), and at a constant temperature of 50 °C, at the lowest ultrasound intensity of 1.62 W/cm^2^, the yield of d-Limonene recovered was found to be 8.97 mg/100 g of dry CPW and at the highest ultrasound intensity of 49.92 W/cm^2^, the yield of d-Limonene was found to be 1.83 mg/100 g of dry CPW. From these results, it is evident that regardless of temperature levels, lower ultrasound intensities result in higher yields of d-Limonene compared to higher intensities. At the highest level of ultrasound intensity, the lowest yield of d-Limonene was recovered.

To investigate the impact of ultrasound as a novel extraction method, a control experiment was carried out with no ultrasound initiated in the extraction and was compared to the experiments with ultrasound in the CCD. The control was carried out at 50 °C for 60 min and the yield of d-Limonene was quantified. The yield of d-Limonene recovered was 64.34 ± 6.91 mg/100 g of dry CPW. When the control experiment was compared to experiments 9 and 10 of the CCD (at the same temperature but with ultrasound applied), it is evident that the application of ultrasound at an intensity of 25.77 W/cm^2^ resulted in lower yields (24.99 and 20.94 mg/100 g of dry CPW respectively). Overall, it can be observed from Fig. 3 that various conditions of ultrasound and temperature have resulted in yields significantly higher or lower compared to the control. The experiments that resulted in higher yields are experiment 1 (25 °C, 8.69 W/cm^2^), experiment 2 (25 °C, 42.85 W/cm^2^) and experiment 5 (14.6 °C, 25.77 W/cm^2^), while the experiments that have resulted in lower yields are experiment 4 (75 °C, 42.85 W/cm^2^), experiment 7 (50 °C, 1.62 W/cm^2^), experiment 8 (50 °C, 49.92 W/cm^2^), as well as experiment 9 and 10 (50 °C, 25.77 W/cm^2^). From these results, it is clear that conditions that have resulted in significant improvement in the extraction of d-Limonene were at lower levels of temperature. Differently, the conditions that have led to significantly less d-Limonene recovery compared to the control are at a combination of higher levels of temperature and ultrasound.

#### Optimum and model validation

The optimum conditions for recovering (removing) d-Limonene were determined at the endpoint of extraction using desirability profiles on the reduced model of Eq. (3). In order to reduce the model, significant factors (*p* < 0.05) and those that were slightly insignificant (*p* >  ~ 0.05) were included in the model. Insignificant factors were pulled into the error term of the model. The reduced model is provided by Eq. (4) below.

4 $$Y=22.87-55.54{X}_{1}-24.73{X}_{2}+78.88{X}_{1}^{2}$$

The optimized level of process variables of 14.6 °C and 25.81 W/cm^2^ resulted in the highest removal yield of d-Limonene, the predicted yield of d-Limonene was 141 mg d-Limonene /g dry CPW with a desirability value of 1. The following results are crucial as they suggest that this pre-treatment stage using ultrasound for the removal of d-Limonene can be achieved at relatively low temperatures. To validate the model, conditions of 14.6 °C and 25.81 W/cm^2^ were used as the appropriate pre-treatment conditions at which the yield of d-Limonene recovered was predicted to be 141.27 mg/100 g dry CPW. At these conditions, the validation run gave a yield of 134 ± 4.24 mg/100 g dry CPW. These pretreatment conditions were then used for the treatment of the CPW prior to fermentation investigations.

#### Effect of ultrasound pre-treatment on d-Limonene removal and bioethanol production. 

**Fig. 4:**
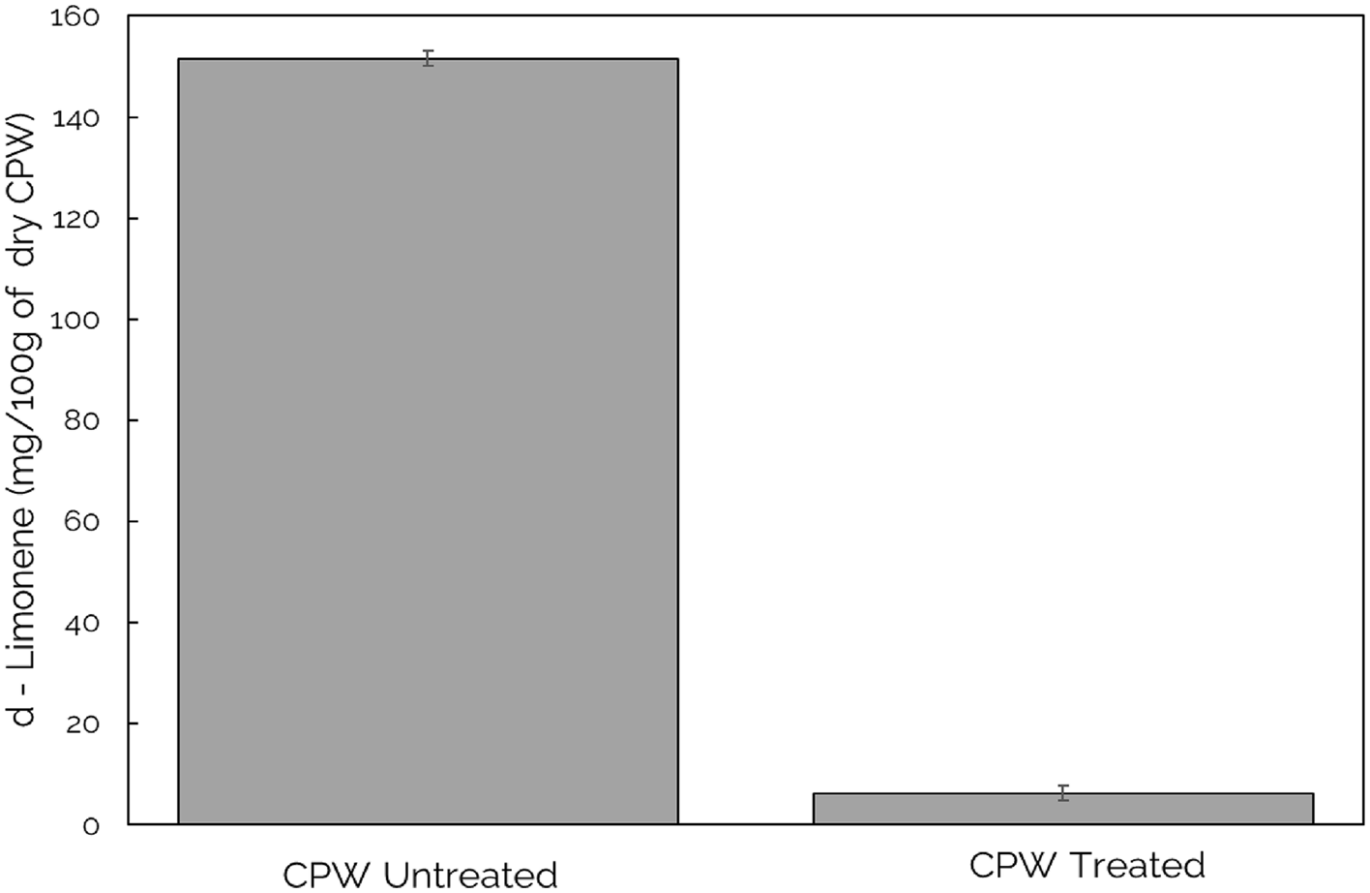
Concentration of d-Limonene in citrus peel waste (ultrasound treated and untreated. CPW-citrus peel waste before SSF (Simultaneous saccharification and fermentation) and SHF (Sequential hydrolysis and fermentation)

To understand the influence of the optimized ultrasonication pre-treatment strategy on the final production of bioethanol, two bioethanol production routes were investigated i.e., simultaneous saccharification and fermentation (SSF) and sequential hydrolysis and fermentation (SHF). Before bioethanol production, d-Limonene, the essential oil component in CPW was investigated either treated (using ultrasound technique) or untreated (as raw peels). From Fig. 4, untreated CPW contained 151.54 $\pm$ 1.47 of d-Limonene yield before SSF and SHF processes, as compared to the reduced yield of 6.27 $\pm$ 1.19 after ultrasound treatment.

To set up bioethanol production, 180 g of the CPW slurry treated and untreated was used for SSF and SHF. As the d-Limonene yield, similar results are reported in Table 4 for bioethanol production from CPW. From an unoptimized bioethanol production procedure of the SSF and SHF, Table 4 shows a 66% increase can be spotted in bioethanol production between the ultrasound treated and the untreated CPW during SSF, and a 29% difference was also seen during the SHF process. Interestingly, a 29% increase was spotted when comparing the SSF and SHF results, which was not surprising as a similar trend is shown in the literature between SSF and SHF bioethanol production [30].

**Table 4:** The effect of ultrasound pre-treatment on bioethanol production,—SSF (Simultaneous saccharification and fermentation) and SHF (Sequential hydrolysis and fermentation), Con–concentration

Content	SHF (g/L)	SSF (g/L)
Untreated (Con/180 g of CPW)	0.94 $\pm$ 0.06	0.85 $\pm$ 0.22
Treated (Con/180 g of CPW)	1.39 $\pm$ 0.19	1.97 $\pm$ 0.12

### Discussions

The present study aimed to access the technical viability of utilizing ultrasonication for treating CPW (with the goal of removing essential oils, d-Limonene) in aqueous systems, followed by subsequent fermentation for bioethanol fermentation. The initial focus was on identifying optimal conditions for d-Limonene recovery through ultrasonication, as presented in the results section above. The findings revealed significant insights into the relation between temperature, ultrasound intensity and d-Limonene extraction, shedding a light on the underlying cavitational phenomena. The study further validated the application of optimized ultrasonication for d-Limonene removal toward bioethanol production, highlighting its practicality toward improved bioethanol production.

The influence of temperature and ultrasound intensity on d-Limonene extraction was a key aspect of this study. It was found that ultrasonication at lower temperatures favored the extraction of d-Limonene, as higher yields where obtained. This result was expected as ultrasound cavitation is more violent at lower temperatures than at higher temperatures. Since vapor pressure is known to increase with temperature, at low temperatures, vapor pressure is lower, and ultrasound produces fewer bubbles; however, the bubbles explode with greater force due to the big difference between the pressure inside and outside bubbles. At higher temperatures, bubbles are produced more readily, however, these bubbles collapse with less intensity due to smaller differences in pressures, the less intense cavitation probably led to the low yields as earlier reported [31]. Similar trends were found by studies conducted by Zhang et al. (2008), in which the essential oil yield from flaxseed decreased by 6% as temperature was increased from 30 to 50 ºC, attributed to decreased cavitation effects at higher temperatures. Also, Khandere et al. (2021) recently highlighted the influence of temperature on the extraction of d-Limonene during ultrasonication [32]. Hence, the effect spotted in this study necessitates an investigation on temperature parameter should essential oil extraction be of paramount importance.

As temperature influences essential oil extraction, ultrasound intensity is also a key factor during essential oil extraction since it directly affects the procedure by affecting the cavitational effects. From this study, the decrease of d-Limonene yield with increasing ultrasound intensity was not expected as the cavitational effects (mechanical and thermal) of ultrasound are expected to increase with increasing intensity. It is known that increasing ultrasound intensity leads to the transmission of a large number of bubbles with more rapid and violent collapse, as the bubbles collapse within the vicinity of plant matter, the shear forces generated lead to micro-fractures and disintegration of cell organelles, which ultimately leads to washing and release of intracellular content of the plant material [33, 34]. However, in this study, it is likely that the decrease in the yield of d-Limonene was due to the decomposition of the oil under the high shear, high localized temperatures and high free radicals generated at high intensities. Khandere et al. (2021) discovered that when ultrasonic intensity was increased from 0.21 to 0.84 W/cm^2^, the yield of d-Limonene extracted increased. However, a further rise from 0.84 to 0.98 W/cm^2^ resulted in a drop in d-Limonene yield. According to the authors, it is possible that the oil degraded as a result of increased cavitation effects [32]. The intensities in our study are considerably higher, implying that it is plausible that the higher intensities have led to oil decomposition, even more so when compared to a process when ultrasound is not applied. Various studies have also highlighted the impact of ultrasound intensity on yield of essential oils [31, 33, 35]. In studies by Chen et al., (2021) and Zhang et al., (2008), increase in ultrasound intensity was found to increase the yield of the extracted oil, with no decline observed [31, 33]. Noteworthy though, in the study by Chen, essential oils were extracted from cinnamon barks, while the study by Zhang, the essential oils were extracted from flaxseeds. Cinnamon barks and flaxseeds cell walls are recalcitrant, of which ultrasound disruptions would aid in mass transfer and thus recovery of essential oils. Differently, CPW cell wall is not recalcitrant, permeating easier access to the oils. The differences in essential oil extraction behavior highlights that ultrasound systems needs to be optimized for specific substrates toward optimal extractions [36, 37].

Differently, under high intensities, it is expected that the cavitation effect decreases leading to decreased yields. The decline of cavitational effects with increasing intensity is attributed to the formation of more bubbles at high intensity which lowers the transmission of ultrasound waves to the medium. However, a calometric study used to determine the ultrasound intensities in this study indicated that at different nominal ultrasound power (30, 50, 100, 150 and 170 W) at a constant frequency (24 kHz), the dissipated power was 0.62, 3.34, 9,92, 16.49 and 19.21 W respectively. The ultrasound intensities for these dissipated powers were calculated to be 1.63, 8.69, 25.77, 42.85, and 49.92 W/cm^2^. The results from the calorimetric study show that it is less likely that the transmission of ultrasound decreasing at high intensity caused the decline in the yield, but rather the high shear, higher localized temperatures and interaction with free radicals had led to the decomposition of the oil. Thus, from this study, temperature of 14.6 °C and ultrasound intensity of 25.81 W/cm^2^ were determined to be the optimum at which CPW was treated to remove d-Limonene before fermentations.

From the results in Fig. 4, it is evident that ultrasound pre-treatment aids in reducing the d-Limonene present in the CPW. This is possible due to the ultrasound mechanical effects that disrupt the CPW cell walls, thereby facilitating mass transfer through an improved penetration of solvents into the cells for essential oil removals [21, 32, 37–39]. Hence, the expected drop from untreated to treated is anticipated due to the acoustic cavitation presented by this pre-treatment technique.

The possibility of treating the CPW implies a limited amount of the essential oil (d-Limonene) will be present during fermentation, as shown in Table 4. This means the oil’s antimicrobial properties limit fermentation by damaging the membrane of the *S. cerevisiae,* and if that is reduced before fermentation, a better yield of bioethanol can be recorded. Hence, this explains the 66 and the 29% increase in bioethanol yield between treated and untreated CPW during SSF and SHF respectively. These trends are similar to those shown in literature for different pre-treatment techniques in mandarin and orange peels. They (pre-treatment techniques) aid in providing accessibility between the substrate and the enzyme used, thereby, an increased yield is expected [22–24, 40, 41].

Finally, a 29% increase between the SSF and SHF was spotted in the treated CPW bioethanol production, which is the case with other lignocellulosic biomass in bioethanol production reported [30, 40, 42]. These changes result from less inhibition of the catalyst (enzyme) used after pre-treatment hydrolysate, leading to better fermentation yield. Although, the fermentation yield in terms of bioethanol concentration is low for either SHF or SSF, several factors such as producing micro-organism or concentration of the sugar in the CPW could be better investigated to increase bioethanol yield. Hence, this process could be advised in CPW bioethanol production by incorporating the ultrasound and later bioethanol production followed by biorefinery techno-economic feasibility study.

## Conclusions

This study aims at utilizing an ultrasound-assisted extraction technique as a novel pre-treatment method for the extraction of d-Limonene (essential oil from citrus peel waste) followed by investigating its effect on bioethanol production from citrus peel waste. The functionality of this technique led to an optimization study on d-Limonene, followed by an investigation of its effect (with or without d-Limonene) during fermentation with several key insights demonstrated below:

- *The optimum effect of pre-treatment* – based on the pre-treatment parameters of temperature and ultrasound intensity on the yield of extracted d-Limonene, optimum values of 14.6 °C and 25.81 W/cm^2^ were obtained respectively. These values imply that ultrasonication cavitation promotes the release of d-Limonene (from CPW, *Citrus clementina*) preferably at lower temperatures and lower ultrasound intensities. Hence, it is required for these parameters to be investigated when considering different plants toward optimum essential oil removal.
- *Yield improvement* – based on the pre-treated and untreated strategy of the citrus peel waste, a 66 and 29% yield increment during SSF and SHF for bioethanol fermentation processes was reported respectively. These values indicate a significant need for a pre-treat strategy (such as ultrasonication) to be employed for waste materials that contain essential oil in the cell wall before fermentation. Hence, proper optimization of the bioethanol production step is important should this process be valued for a biorefinery implementation.

## Data Availability

The data generated and analysed during this study is available from the corresponding authors on reasonable request.

## References

[1] González-Molina E, Domínguez-Perles R, Moreno DA, García-Viguera C (2010). Natural bioactive compounds of citrus limon for food and health. J Pharm Biomed Anal.

[2] Perez-Perez JG, Castillo IP, Garcia-Lidon A (2005). Fino lemon clones compared with the lemon varieties Eureka and Lisbon on two rootstocks in Murcia (Spain). Sci Hortic.

[3] Hamdan D, El-Readi MZ, Nibret E (2010). Chemical composition of the essential oils of two citrus species and their biological activities. Pharmazie.

[4] Russo C, Maugeri A, Lombardo GE (2021). The second life of citrus fruit waste: a valuable source of bioactive compounds. Molecules.

[5] Chavan P, Singh AK, Kaur G (2018). Recent progress in the utilization of industrial waste and by-products of citrus fruits: a review. J Food Process Eng.

[6] Wadhwa M (2013). Bakshi MPS (2013) Utilization of fruit and vegetable wastes as livestock feed and as substrates for generation of other value-added products. Rap Publ.

[7] Martín MA, Siles JA, Chica AF, Martín A (2010). Biomethanization of orange peel waste. Bioresour Technol.

[8] David J, Medina C, Magalhaes AI (2012). chapter ethanol production current facts, future scenarios, and techno-economic assessment of different biorefinery configurations. Bioethanol Technol.

[9] Bakkali F, Averbeck S, Averbeck D, Idaomar M (2008). Biological effects of essential oils – a review. Food Chem Toxicol.

[10] Choi IS, Lee YG, Khanal SK (2015). A low-energy, cost-effective approach to fruit and citrus peel waste processing for bioethanol production. Appl Energy.

[11] Zhao S, Zhang D (2014). Supercritical CO2 extraction of Eucalyptus leaves oil and comparison with Soxhlet extraction and hydro-distillation methods. Sep Purif Technol.

[12] Wang L, Weller CL (2006). Recent advances in extraction of nutraceuticals from plants. Trends Food Sci Technol.

[13] Teke GM, Pott RWM (2021). Design and evaluation of a continuous semipartition bioreactor for in situ liquid-liquid extractive fermentation. Biotechnol Bioeng.

[14] Teke GM, Gakingo GK, Pott RWM (2022). The liquid-liquid extractive fermentation of L–lactic acid in a novel semi-partition bioreactor (SPB). J Biotechnol.

[15] Teke GM, Tai SL, Pott RWM (2022). Extractive fermentation processes: modes of operation and application. ChemBioEng Rev.

[16] Teke GM, Gakingo GK, Pott RWM (2022). Towards improved understanding of the hydrodynamics of a semi-partition bioreactor (SPB): a numerical investigation. Chem Eng Res Des.

[17] Teke GM, Gakingo GK, Pott RWM (2022). A Numerical investigation of the hydrodynamic and mass transfer behavior of a liquid- liquid semi-partition bioreactor ( SPB ) designed for in-situ extractive fermentation. Chem Eng Sci.

[18] Teke GM (2022) Design and investigation of a semi-partitioned bioreactor (SPB) for extractive fermentation using computational fluid dynamic (CFD) simulations and experimental studies. PhD Dissertation. Stellenbosch Univeristy

[19] Singh B, Singh JP, Kaur A, Yadav MP (2021). Insights into the chemical composition and bioactivities of citrus peel essential oils. Food Res Int.

[20] Farhat A, Fabiano-Tixier AS, El MM (2011). Microwave steam diffusion for extraction of essential oil from orange peel: Kinetic data, extract’s global yield and mechanism. Food Chem.

[21] Quintero Quiroz J, Naranjo Duran AM, Silva Garcia M (2019). Ultrasound-assisted extraction of bioactive compounds from annatto seeds, evaluation of their antimicrobial and antioxidant activity, and identification of main compounds by LC/ESI-MS analysis. Int J Food Sci.

[22] Santi G, Crognale S, D’Annibale A (2014). Orange peel pretreatment in a novel lab-scale direct steam-injection apparatus for ethanol production. Biomass Bioenergy.

[23] Wilkins MR, Widmer WW, Grohmann K (2007). Simultaneous saccharification and fermentation of citrus peel waste by Saccharomyces cerevisiae to produce ethanol. Process Biochem.

[24] Choi IS, Kim JH, Wi SG (2013). Bioethanol production from mandarin (*Citrus unshiu*) peel waste using popping pretreatment. Appl Energy.

[25] Qiu J, Shi M, Li S (2023). Artificial neural network model- and response surface methodology-based optimization of Atractylodis Macrocephalae Rhizoma polysaccharide extraction, kinetic modelling and structural characterization. Ultrason Sonochem.

[26] Wang X, Liu X, Shi N (2023). Response surface methodology optimization and HPLC-ESI-QTOF-MS/MS analysis on ultrasonic-assisted extraction of phenolic compounds from okra (*Abelmoschus esculentus*) and their antioxidant activity. Food Chem.

[27] AtwiGhaddar S, Destandau E, Lesellier E (2023). Optimization of supercritical fluid extraction of polar flavonoids from *Robinia pseudoacacia* L. heartwood. J CO2 Utilization.

[28] Bagade SB, Patil M (2021). Recent advances in microwave assisted extraction of bioactive compounds from complex herbal samples: a review. Crit Rev Anal Chem.

[29] Titiri E, Filippi K, Giannakis N (2023). Optimisation of alkaline pretreatment of spent coffee grounds for microbial oil production by Cryptococcus curvatus. Biochem Eng J.

[30] Dahnum D, Tasum SO, Triwahyuni E (2015). Comparison of SHF and SSF processes using enzyme and dry yeast for optimization of bioethanol production from empty fruit bunch. Energy Procedia.

[31] Zhang ZS, Wang LJ, Li D (2008). Ultrasound-assisted extraction of oil from flaxseed. Sep Purif Technol.

[32] Khandare RD, Tomke PD, Rathod VK (2021). Kinetic modeling and process intensification of ultrasound-assisted extraction of d-Limonene using citrus industry waste. Chem Eng Process Process Intensif.

[33] Chen G, Sun F, Wang S (2021). Enhanced extraction of essential oil from *Cinnamomum cassia* bark by ultrasound assisted hydrodistillation. Chin J Chem Eng.

[34] Li H, Pordesimo L, Weiss J (2004). High intensity ultrasound-assisted extraction of oil from soybeans. Food Res Int.

[35] Chen F, Liu S, Zhao Z (2020). Ultrasound pre-treatment combined with microwave-assisted hydrodistillation of essential oils from *Perilla frutescens* (L.) Britt. leaves and its chemical composition and biological activity. Ind Crops Prod.

[36] Siddiqui SA, Pahmeyer MJ, Assadpour E, Jafari SM (2022). Extraction and purification of d-Limonene from orange peel wastes: recent advances. Ind Crops Prod.

[37] Ferreira IJB, Alexandre EMC, Saraiva JA, Pintado M (2022). Green emerging extraction technologies to obtain high-quality vegetable oils from nuts: a review. Innovative Food Sci Emerg Technol.

[38] Sharma K, Mahato N, Cho MH, Lee YR (2017). Converting citrus wastes into value-added products: economic and environmently friendly approaches. Nutrition.

[39] Tekin K, Akalin MK, Şeker MG (2015). Ultrasound bath-assisted extraction of essential oils from clove using central composite design. Ind Crops Prod.

[40] Wyman CE, Spindler DD, Grohmann K (1992). Simultaneous saccharification and fermentation of several lignocellulosic feedstocks to fuel ethanol. Biomass Bioenergy.

[41] Widmer W, Zhou W, Grohmann K (2010). Pretreatment effects on orange processing waste for making ethanol by simultaneous saccharification and fermentation. Bioresour Technol.

[42] Öhgren K, Bura R, Lesnicki G (2007). A comparison between simultaneous saccharification and fermentation and separate hydrolysis and fermentation using steam-pretreated corn stover. Process Biochem.

